# Activity in the brain’s valuation and mentalizing networks is associated with propagation of online recommendations

**DOI:** 10.1038/s41598-021-90420-2

**Published:** 2021-05-27

**Authors:** Elisa C. Baek, Matthew Brook O’Donnell, Christin Scholz, Rui Pei, Javier O. Garcia, Jean M. Vettel, Emily B. Falk

**Affiliations:** 1 Annenberg School for Communication, University of Pennsylvania, 3620 Walnut St., Philadelphia, PA 19104 USA; 2 Amsterdam School of Communication Research, University of Amsterdam, Amsterdam, The Netherlands; 3 US DEVCOM, Army Research Laboratory, Adelphi, USA; 4 Department of Bioengineering, University of Pennsylvania, Philadelphia, USA; 5 Department of Psychological Brain Sciences, University of California, Santa Barbara, Santa Barbara, USA; 6 Department of Psychology, University of Pennsylvania, Philadelphia, PA 19104 USA; 7 Marketing Department, The Wharton School, University of Pennsylvania, Philadelphia, PA 19104 USA; 8 Present Address: Department of Psychology, University of California, Los Angeles, Los Angeles, USA

**Keywords:** Social neuroscience, Social behaviour, Human behaviour

## Abstract

Word of mouth recommendations influence a wide range of choices and behaviors. What takes place in the mind of recommendation receivers that determines whether they will be successfully influenced? Prior work suggests that brain systems implicated in assessing the value of stimuli (i.e., subjective valuation) and understanding others’ mental states (i.e., mentalizing) play key roles. The current study used neuroimaging and natural language classifiers to extend these findings in a naturalistic context and tested the extent to which the two systems work together or independently in responding to social influence. First, we show that in response to text-based social media recommendations, activity in both the brain’s valuation system and mentalizing system was associated with greater likelihood of opinion change. Second, participants were more likely to update their opinions in response to negative, compared to positive, recommendations, with activity in the mentalizing system scaling with the negativity of the recommendations. Third, decreased functional connectivity between valuation and mentalizing systems was associated with opinion change. Results highlight the role of brain regions involved in mentalizing and positive valuation in recommendation propagation, and further show that mentalizing may be particularly key in processing negative recommendations, whereas the valuation system is relevant in evaluating both positive and negative recommendations.

## Introduction

Word of mouth recommendations are a powerful form of communication^1^, influencing consumer decisions^2^, political mobilization^3^, and the subjective value of objects and ideas in a wide range of contexts^4–6^. What takes place in the mind of receivers exposed to recommendations from peers, experts, and even strangers that determines the likelihood that the communicator’s opinion spreads further? In the current study, we studied recommendations from peers as one source of social influence on behavior^7^. Past research has suggested that assessing the value of different stimuli (i.e., subjective valuation)^5,6,8–10^ and understanding others’ mental states (i.e., mentalizing)^9–11^ are key processes in adopting and propagating recommendations. These processes are associated with specific networks in the brain: (1) the valuation system, which includes ventromedial prefrontal cortex (VMPFC) and ventral striatum (VS)^12^, and (2) the mentalizing system, which includes portions of the medial prefrontal cortex (MPFC), particularly subregions in the middle and dorsomedial prefrontal cortex (MMPFC, DMPFC), as well as bilateral temporoparietal junction (TPJ), precuneus (PC/PCC), superior temporal sulcus (STS), and temporal poles^13,14^. We used neuroimaging and natural language classifiers: (1) to test the role of these neural systems in updating opinion in response to positive and negative recommendations, (2) to extend past results to a more naturalistic context (i.e., responding to real written recommendations with natural language text), and (3) to examine a new question about the extent to which these neural systems work together or independently to produce recommendation rating change in response to naturalistic recommendations.

### Brain activity in the valuation system predicts successful social influence

Prior neuroimaging research has highlighted the involvement of the brain’s valuation system in successful social influence, supporting the propagation of ideas between a communicator and a receiver (for reviews see^15,16^). Generally, the brain’s valuation system, including the ventral striatum (VS) and the ventromedial prefrontal cortex (VMPFC), computes the subjective value of different types of stimuli, including primary (e.g., food) and secondary (e.g., social) rewards^12^. In the context of social influence on recommendations, the value system is implicated in tracking the value of different decision-relevant information over time, including the social rewards of being in alignment with a group^8,9^, positive valuation of the social recommendation and anticipated rewards of conforming^9–11^, and one’s internal value of the stimuli^5,6,8^.

In the context of online media platforms, people often encounter recommendations that are different from their own opinions, which lead them to update and share their own recommendations. In lab situations paralleling this online social context, the valuation signal in the brain tracks the value of peer recommendations, where greater activity in the valuation system is associated with receivers of influence conforming to peer recommendations versus resisting peer influence^10,11^. Extant neuroimaging studies whose timing most closely mirrors online recommendation contexts (in presenting recommendations and then recording participants’ updated opinions immediately), however, have focused primarily on adolescents^10,11^. This makes it unclear whether these findings are specific to adolescents or more generally true of the process of incorporating peer feedback on recommendations in real-time. In the current neuroimaging study, we tested this paradigm in a young adult sample. If neural signal in the valuation system tracks the value of social recommendations and anticipated rewards of conforming, we hypothesized that increased activation in response to either positive or negative recommendations should track with the participant subsequently updating their opinion in line with peer recommendations.

### Brain activity in the mentalizing system predicts successful information propagation

Prior studies of individual differences in recommenders also offer preliminary evidence suggesting the importance of the brain’s mentalizing system for the successful propagation of ideas (for a review, see^17^). Successful recommenders often show greater neural activity in the mentalizing system compared to less influential recommenders^18–20^. Furthermore, ideas that people want to share elicit greater activity in the mentalizing system^19,21,22^. In parallel, receivers who are more persuadable to update their own recommendations also show greater mentalizing activity^10^, and increased brain activity in mentalizing regions during social feedback is associated with greater likelihood of conforming to peer opinion^11^. Greater activity in mentalizing regions is also observed in the processing of divergent peer influence, including when a receiver of social influence finds out that he or she is not in alignment with peer opinions^9,10^. This finding suggests that the mentalizing system may enable the receiver to understand the recommender’s intentions or point-of-view^9,10^. Thus, the tendency to consider other people’s mental states may be an important element in updating one’s own initial opinion, and we expect that neural activity in the mentalizing system will track the successful spread of recommendations.

### Recommendation valence

Our research also examines an open question in the literature about whether brain activity tracking social influence is sensitive to the valence of recommendations. Mentalizing may broadly aid in understanding others’ viewpoints, and the value system might broadly assess the value of peer recommendations, tracking with opinion change in response to both positive and negative recommendations; alternatively, these systems may respond more strongly in situations where people are most likely to assess social consequences of their actions, such as in response to negative social evaluation^23–26^. Behavioral evidence suggests that negative (versus positive) peer recommendations may lead to greater conformity^2,10^. This ‘negativity bias’, or the idea that people exhibit greater sensitivity to negative information than positive information of equal objective polarity, has been observed across diverse fields^27^. To this end, we tested whether the valence of peer recommendations influences the engagement of the valuation and mentalizing systems during recommendation propagation.

### Does brain connectivity between valuation and mentalizing systems predict conformity or resistance to peer influence on recommendations?

Prior studies of recommendation behavior have focused on average neural activation within specific, separate brain regions (e.g., regions within the valuation and mentalizing systems), and, as such, do not provide insight about how different brain systems might coordinate to facilitate or suppress receptivity to social influence. Therefore, we extend prior work by also examining the interplay between regions of the brain’s valuation and mentalizing systems in recommendation propagation. We tested two competing hypotheses. One possibility is that increased coordination between activity in the brain’s valuation and mentalizing systems in response to peer recommendations might be associated with greater recommendation rating change. If increased value placed on the peers’ opinion leads to greater mentalizing, we anticipate that greater connectivity between these systems would be associated with more recommendation-congruent opinion change. An alternative hypothesis is that increased coordination in activity between the brain’s valuation and mentalizing systems in response to peer recommendations might be associated with *less* recommendation-congruent opinion change. If decreased value placed on the peers’ opinion leads to suppression of mentalizing, we would also anticipate that greater connectivity between these systems could be associated with less recommendation-congruent opinion change. This would be consistent with past research on motivated reasoning^28^. Accordingly, recommendation rating change might be supported by decreased functional connectivity between the brain’s valuation and mentalizing regions. To test these competing hypotheses, we used psychophysiological interaction (PPI) analysis^29^ to compare functional connectivity between the brain’s valuation and mentalizing regions when participants changed (vs. didn’t change) their recommendations in response to peer recommendations. PPI captures the interaction between psychological variables (in the case of this investigation, whether a participant is persuaded to change their recommendation or not) and brain response (in this case, the correlation between activity in the mentalizing and valuation systems). We use this method to determine whether brain responses in mentalizing and valuation systems are more or less correlated when participants update, or do not update, their recommendations based on peer feedback.

### The current study

**Figure 1:**
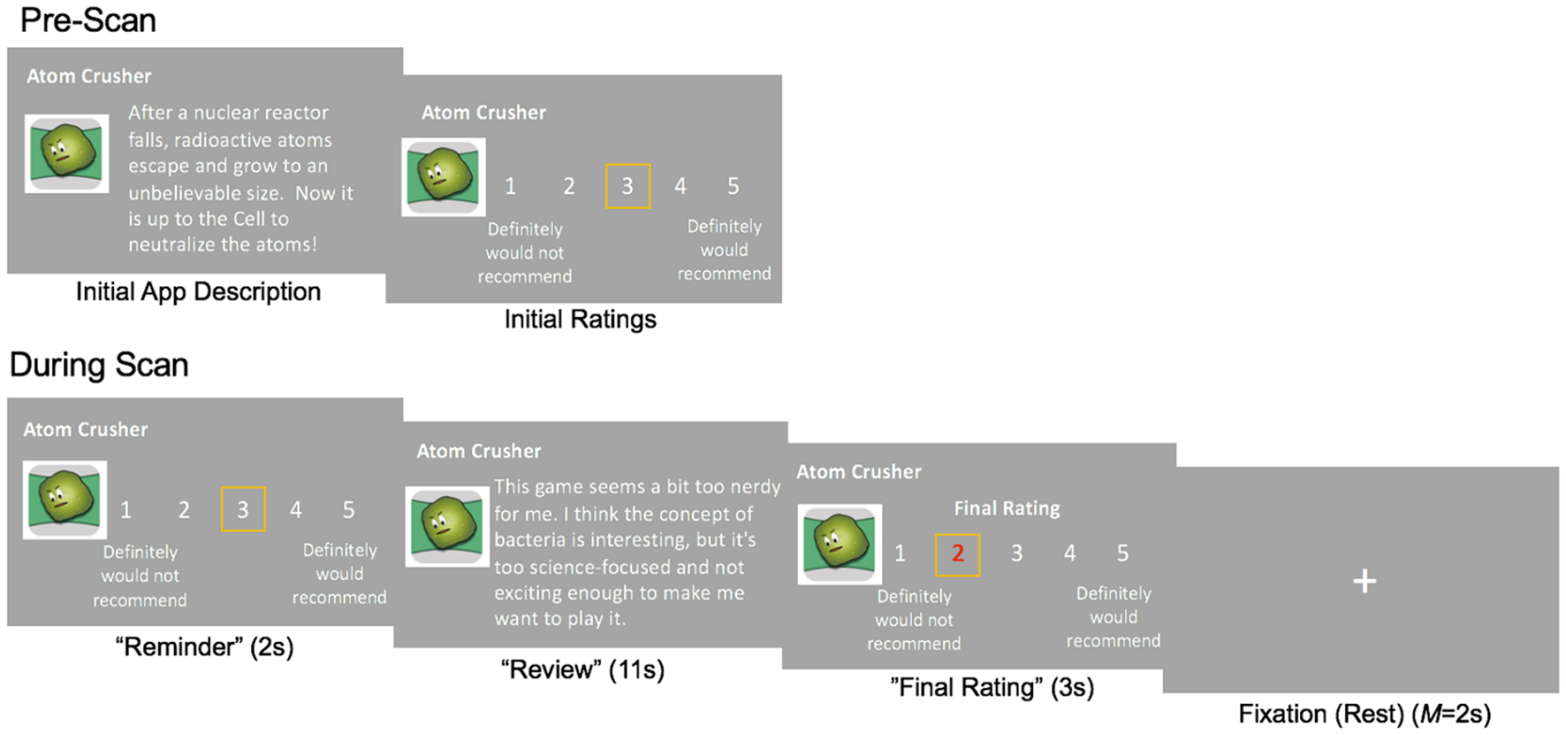
Task Schema. Before the scan, participants saw descriptions of 80 mobile game apps and provided initial ratings of their likelihood to recommend each app to others. In the scanner, participants were first reminded of their initial recommendation ratings. Then, they read peer recommendations about each of the 80 mobile game apps. Recommendations ranged in valence, with some being positive and others negative. During the final rating period of the scan, participants had the opportunity to update their recommendation ratings based on the peer feedback.

Participants performed a modified version of the App Recommendation Task (Cascio et al.^10^; Fig. 1) in which they learned about mobile game apps and then read real text of peer recommendations related to the apps while their brain activity was measured using neuroimaging (functional MRI, or fMRI). The task simulated real-life situations when people consider others’ recommendations during decisions to consume and recommend a product to other people. Before the fMRI scan, participants rated their likelihood to recommend 80 mobile game applications based only on the information from the app developers. Approximately an hour later, during the fMRI scan, participants then read peer recommendations that were written by other users about the same mobile game applications and were given the opportunity to update their own recommendation rating. The valence of the peer recommendations that were shown to participants was scored using a sentiment analysis tool (http://text-processing.com/api/sentiment/), where high scores indicated positivity and low scores indicated negativity. We calculated ‘recommendation rating change’ as being positive if participants changed their own recommendation ratings in the direction of the peer recommendations (i.e., became more positive in response to positive reviews or more negative in response to negative reviews).

Our paradigm also allowed us to test the neural and psychological processes that are implicated in the current online recommendation context, where people are exposed to peer opinions and then update their own recommendations in real-time. Further, we used written recommendations from a separate group of participants, which more closely reflects real-life social influence contexts and a richer, naturalistic measure of social influence. Collectively, our approach allowed us to test the role of the valuation and mentalizing systems in recommendation propagation, and how the valence of peer endorsements may modulate such effects. In a novel contribution to the field of neuroscience of communication and social influence, we also tested whether functional connectivity between the valuation and mentalizing systems is associated with recommendation propagation.

## Materials and methods

### Participants

Thirty-eight participants (27 females; mean age = 20.9) provided complete data, and four participants provided partial data (see Supplementary informationfor exclusions). The study was approved by and conducted in accordance with relevant guidelines and regulations by the Institutional Review Board of the University of Pennsylvania, and all participants gave informed consent for the study procedure.

### Procedure

Participants completed the first part of a modified version of the App Recommendation Task^10^ before the fMRI scan. They read the title, logo, and description of 80 mobile game applications taken from the iTunes App Store and indicated their initial likelihood of recommending (‘initial recommendations’) each game app. In the second part of the task, which took place inside the MRI scanner, participants reviewed the same 80 mobile game apps. For each mobile game app, participants were first shown the title and logo of each game and reminded of their initial recommendation rating for 2 s (‘reminder period’). Then, the participants read a short recommendation of the game app that they were (truthfully) told was written by their peers (*M* = 32.4 words, SD = 7.2 words) for 11 s (‘review period’). The peer recommendations were written by a different group of participants that were similar in demographics to the current study (N = 43, age *M* = 22.1) as part of a separate study. We used text written by a different group of participants to maximize external validity, reflecting real-life online recommendation environments. Each participant read recommendations for 80 mobile game apps, each from one of two pseudo-randomly assigned peer reviewers (one recommendation per app; 40 total recommendations per recommender) (see Supplementary information for more information). After reading each recommendation, participants had 3 s to provide a final rating of their own likelihood to recommend the game app (‘final rating period’). See Fig. 1 for an illustration of the task design.

### fMRI image acquisition

Neuroimaging data from participants were obtained using 3 T Siemens scanners. For each participant, we acquired three functional runs (500 volumes per run) using T2*-weighted reverse spiral sequence (TR = 1500 ms, TE = 25 ms, 54 axial slices, flip angle = 70°, − 30° tilt relative to AC-PC line, FOV = 200 mm, slice thickness = 3 mm; voxel size = 3.0 × 3.0 × 3.0 mm, order of slice acquisition: interleaved). T1-weighted images (MPRAGE; magnetization-prepared rapid-acquisition gradient echo) were recorded (TI = 1110 ms, 160 slices, FOV = 240 mm, slice thickness = 1 mm, voxel size = 0.9 × 0.9 × 1 mm). In-plane structural T2-weighted images were also collected (slice thickness = 1 mm, 176 sagittal slices, voxel size = 1 mm × 1 mm × 1 mm) for use in coregistration and normalization.

### Sentiment analysis

Each peer recommendation that participants read was scored using a sentiment analysis API (http://text-processing.com/api/sentiment/) on a continuous measure of positive to negative sentiment, with the highest score indicating the highest amount of positivity (sentiment = 1.0) and the lowest score indicating the highest amount of negativity (sentiment = 0.0). For example, the recommendation “This game sounds awesome” receives a positive probability score of 0.7, whereas the recommendation “This game sounds terrible” receives a positive probability score of 0.2 (see Table 1 for additional examples). These probability scores from the machine learning classifier represent the conditional probability of the recommendation being positive based on the features occurring in the text.

**Table 1:** Example of peer recommendations and their sentiment scores.

Peer recommendation	Sentiment score
This game was one of the most boring games I have ever played. The idea is not original and the graphics are not what they could be at all. I would save your time	0.10
This bear can only move while touching blocks; so be sure to get rid of all the circles and triangles so she can freely move along to the next level!	0.50
In this game you are on a fantastical journey to release a dragon from a book. It is a unique premise and is definitely entertaining. Original and fun	0.82

### Human coding

To validate the sentiment scores from the machine learning classifier, each peer recommendation was also scored by human coders recruited on Amazon’s Mechanical Turk. Each recommendation was rated by 3 human coders on a 0–100 scale (0 = most negative; 100 = most positive) with high interrater reliability (Krippendorff’s alpha = 0.738).

### Behavioral data analysis

To investigate whether peer recommendations influenced participants to change from their initial recommendation rating, we ran a multi-level linear model in R^30^ using the *lme4*^31^ and *lmerTest*^32^ packages. We defined recommendation rating change as being positive (+ 1) if the participant changed their initial ratings in the direction of the sentiment of the peer recommendation, negative (− 1) if the participant changed their initial ratings away from the sentiment of the peer recommendation, and zero (0) if participants did not change their ratings. For this purpose, peer recommendations were classified into binary categories as either “positive” or “negative” by using the probability scores produced by the sentiment analysis; if the classifier indicated that the recommendation was more likely to be positive than negative, then it was categorized as positive (and vice versa). Thus, if participants changed their initial recommendation of a “5” to a final recommendation rating of a “3” after reading a peer recommendation that was classified as “negative”, then the recommendation rating change was calculated as “1”. To determine the relationship between peer recommendation sentiment scores and participants’ recommendation rating change, we ran a mixed effect (i.e., multi-level) linear regression predicting the participants’ recommendation rating change from the sentiment scores of the peer recommendations. Participants and mobile game apps were treated as random effects with intercepts allowed to vary randomly, accounting for non-independence in the data due to repeated measures from each participant and mobile game app:

$${\text{Recommendation rating change}}_{{{\text{ij}}}} = B_{0} + B_{1} {\text{sentiment}}_{{{\text{ij}}}} +\mu _{0i} +\nu _{0j} +\epsilon _{{{\text{ij}}}} ,$$

where *B*_0_ is the overall intercept, representing the grand mean across all observations, *B*_1_ is an unstandardized regression coefficient capturing the average slope of the relationship between sentiment and recommendation rating change; subscript *i* refers to participant, *j* refers to app, and μ_0i_ and ν_0j_ represent the random errors for the deviation of the mean intercept for each participant and app from the grand mean intercept, respectively, and $\epsilon _{{\rm ij}} ,$ is the random error for each app rating within participants.

### Imaging data analysis

Functional data were pre-processed and analyzed using Statistical Parametric Mapping (SPM8, Wellcome Department of Cognitive Neurology, Institute of Neurology, London, UK). To allow for stabilization of the BOLD (blood oxygen level dependent signal), the first five volumes (7.5 s) of each run were not collected. Functional images were despiked using the 3dDespike program as implemented in the AFNI toolbox^33^. Next, data were corrected for differences in the time of slice acquisition using sinc interpolation, with the first slice serving as the reference slice (using FSL Slicetimer^34^). Data were then spatially realigned to the first functional image. Next, in-plane T2-weighted images were registered to the mean functional image. Next, high-resolution T1 images were registered to the in-plane image (12 parameter affine). After coregistration, high-resolution structural images were segmented into gray matter, white matter, and cerebral spinal fluid (CSF) to create a whole brain mask for use in modeling. Masked structural images were normalized to the skull-stripped MNI template provided by FSL (“MNI152_T1_1mm_brain.nii”). Finally, functional images were smoothed using a Gaussian kernel (8 mm FWHM).

### Regions of interest analysis

**Figure 2:**
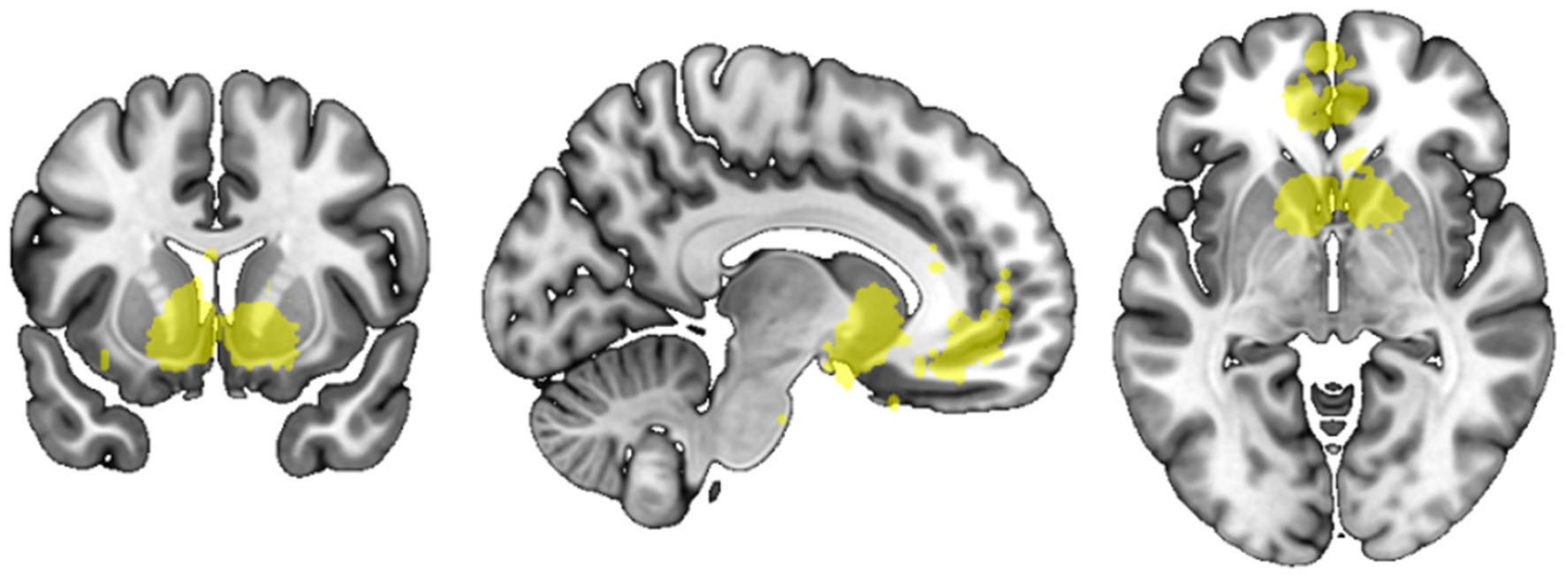
Brain regions associated with “value”, as identified through Neurosynth using an association test, *p* < 0.01, corrected. Figure was created using MRIcro^48^ by the authors.

**Figure 3:**
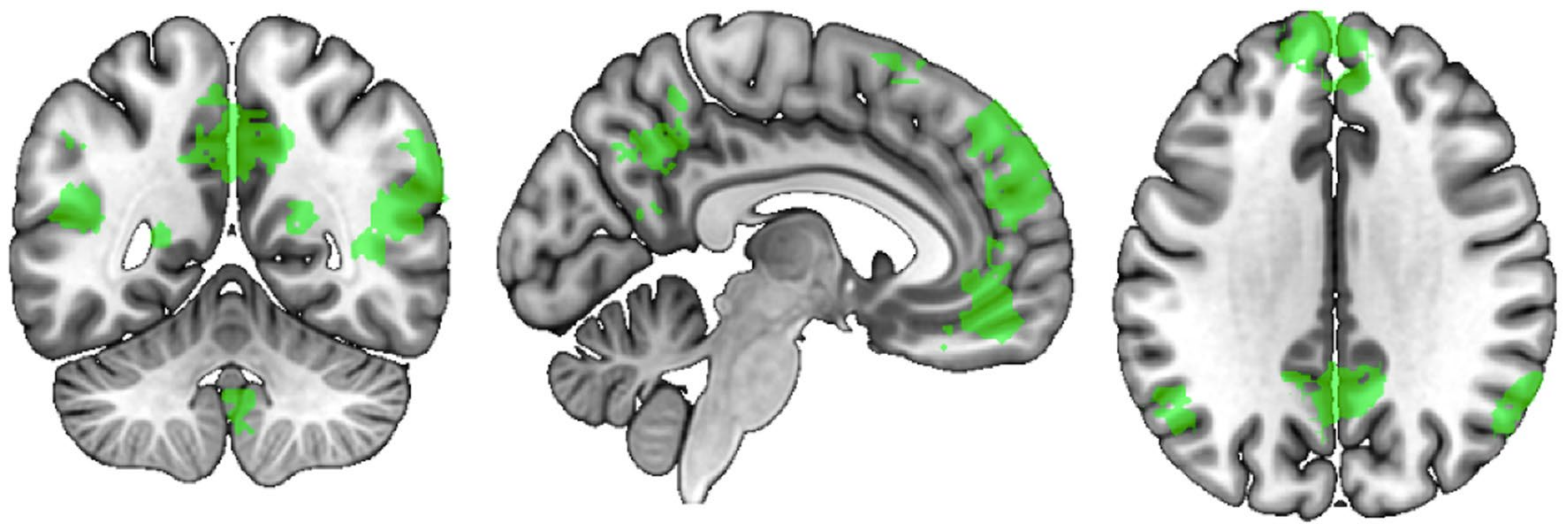
Brain regions associated with “mentalizing”, as identified through Neurosynth using an association test, *p* < 0.01, corrected. Figure was created using MRIcro^48^ by the authors.

We used Neurosynth^35^ to define targeted brain regions of interest. Specifically, we used “association test” meta-analytic maps of the functional neuroimaging literature on “value”, which consisted of subregions in the striatum and ventral medial prefrontal cortex (VMPFC) (see Fig. 2), and “mentalizing”, which consisted of subregions in the middle and dorsal medial prefrontal cortex (MMPFC, DMPFC), bilateral temporoparietal junction (TPJ), precuneus (PC/PCC), middle temporal gyrus (MTG) (see Fig. 3).

### Task and item-based analyses

Data were modeled using the general linear model as implemented in SPM8. For each trial, the review (11 s) and final rating (3 s) periods were modeled together, since participants were incorporating peer recommendations to inform their final recommendation ratings during both periods. All models included six rigid-body translation and rotation parameters derived from spatial realignment as nuisance regressors. Low-frequency noise was removed using a high-pass filter (128 s). We constructed individual models for each subject in which the review and final rating periods for each mobile game app were treated as separate regressors in the design matrix (i.e., an item-based model) using SPM8. Reminder periods across trials were modeled using one regressor of no interest. Fixation periods (i.e., rest periods) served as an implicit baseline. Neural activity in our mentalizing and valuation ROIs was extracted for each mobile game app at the individual level, and percent signal change was calculated by dividing mean task activity by the baseline/rest period. For each participant, the extracted percent signal change was mean centered across the mobile game apps.

### Combining mean brain activity and behavior data

In order to understand the relationship between brain activity and sentiment of peer recommendations, and participants’ recommendation rating change, we ran linear mixed effects models (i.e., multi-level regression models) in R^30^ using the *lme4*^31^ and *lmerTest*^32^ packages. Participants and mobile game app were treated as random effects with intercepts allowed to vary randomly, accounting for non-independence in the data due to repeated measures from each participant and mobile game app.

First, to examine whether neural activity was influenced by the sentiment of peer recommendations participants received in the scanner, we ran multi-level linear regression models predicting participants’ percent signal change in each of our ROIs from the sentiment scores, including random intercepts for participant and app:

$${\text{Mean brain activity}}_{{{\text{ij}}}} = B_{0} + B_{1} {\text{sentiment}}_{{{\text{ij}}}} +\upmu _{0i} +\upnu _{0j} +\upepsilon _{{{\text{ij}}}} ,$$

where *B*_0_ is the overall intercept, representing the grand mean across all observations, *B*_1_ is an unstandardized regression coefficient capturing the average slope of the relationship between sentiment and brain activity; subscript *i* refers to participant, *j* refers to app, and μ_0i_ and ν_0j_ represent the random errors for the deviation of the mean intercept for each participant and app from the grand mean intercept, respectively, and $\epsilon _{{\rm ij}} ,$ is the random error for each app rating within participants; “brain activity” represents activity in the target regions of interest, with separate models run for mentalizing and valuation systems.

Next, to determine the relationship between brain activity and participants’ recommendation rating change, we ran additional multi-level linear regressions predicting participants’ recommendation rating change from neural activity extracted as percent signal change from each of our ROIs per mobile game app, including random intercepts for participant and app:

$${\text{Recommendation rating change}}_{{{\text{ij}}}} = B_{0} + B_{1} {\text{brain activity}}_{{{\text{ij}}}} +\upmu _{0i} +\upnu _{0j} +\upepsilon _{{{\text{ij}}}} ,$$

where *B*_0_ is the overall intercept, representing the grand mean across all observations, *B*_1_ is an unstandardized regression coefficient capturing the average slope of the relationship between brain activity and recommendation rating change; subscript *i* refers to participant, *j* refers to app, and μ_0i_ and ν_0j_ represent the random errors for the deviation of the mean intercept for each participant and app from the grand mean intercept, respectively, and $\epsilon _{{\rm ij}} ,$ is the random error for each app rating within participants; “brain activity” represents activity in the target regions of interest, with separate models run for mentalizing and valuation systems.

Finally, to determine whether the effects of brain activity on recommendation rating change were particularly driven by positive or negative recommendations, we tested the interaction between brain activity and sentiment to predict recommendation rating change, including random intercepts for participant and app:

$${\text{Recommendation rating change}}_{{{\text{ij}}}} = B_{0} + B_{1} {\text{brain activity}} + B_{2} {\text{sentiment}}_{{{\text{ij}}}} + B_{3} {\text{brain activity}}*{\text{sentiment}} +\upmu _{0i} +\upnu _{0j} +\upepsilon _{{{\text{ij}}}} ,$$

where *B*_0_ is the overall intercept, representing the grand mean across all observations, *B*_1_ is an unstandardized regression coefficient capturing the average slope of the relationship between brain activity and recommendation rating change, *B*_2_ is an unstandardized regression coefficient capturing the average slope of the relationship between sentiment and recommendation rating change, *B*_3_ is an unstandardized regression coefficient capturing the average slope of the interaction effect of brain activity and sentiment on recommendation rating change; subscript *i* refers to participant, *j* refers to app, and μ_0*i*_ and ν_0*j*_ represent the random errors for the deviation of the mean intercept for each participant and app from the grand mean intercept, respectively, and $\epsilon _{{\rm ij}} ,$ is the random error for each app rating within participants; “brain activity” represents activity in the target regions of interest, with separate models run for mentalizing and valuation systems. For these analyses, we mean centered the sentiment variable (i.e., so that 0 = neutral sentiment). As previously noted, the brain activity variables were mean centered within each participant for all analyses.

### Psychophysiological interaction (PPI) analysis

We next tested the relationship between functional connectivity between neural activity in the brain’s valuation and mentalizing systems and recommendation rating change. We used psychophysiological interaction (PPI) analysis^29^. PPI tests the hypothesis that brain activity in one region (e.g., mentalizing system) can be explained by the interaction between brain activity in another region (e.g., valuation system) and a cognitive process (e.g., accepting vs. resisting peer influence). Accordingly, we used PPI to compare the strength of functional connectivity between the brain’s mentalizing and valuation systems when participants changed their recommendation ratings to be congruent with peer recommendations (recCHANGE) versus when participants did not change their recommendation ratings (NOrecCHANGE). We used the same valuation region of interest as defined above for the mean activation analyses as the seed region. Using the SPM generalized PPI toolbox^36^, time courses in the seed region were extracted, averaged, and deconvolved with the canonical HRF using the deconvolution algorithm in SPM8 for each participant. Then, the time course in the seed region was multiplied by the behavior variable of interest (recCHANGE vs. NOrecCHANGE), and this resulting time course was re-convolved with the canonical HRF. The PPI model also included 6 motion parameters as nuisance regressors of no interest. The group-level model was then created by combining first-level contrast images using a random effects model. Finally, average parameter estimates of functional connectivity between the seed (i.e., valuation) region and target mentalizing region of interest were extracted at the group level. We then conducted a t-test for statistical inference, to determine whether the extracted parameter estimate was significantly different than zero at *p* < 0.05.

## Results

Our analysis examined whether the brain’s mentalizing and valuation systems could account for variability in changing participants’ own recommendations in response to peer recommendations. We related mean brain activity in the valuation and mentalizing systems to (1) the sentiment of the peer recommendations, (2) whether participants changed their original recommendations after reading peer recommendations (i.e., recommendation rating change), and (3) the interaction of the mean brain activity in the valuation and mentalizing systems with the sentiment of the peer recommendations to predict recommendation rating change. We then also tested whether functional connectivity between the valuation and mentalizing systems was associated with increased or decreased likelihood of recommendation rating change.

### Sentiment classifier and human coding

We first compared our machine learning classifier sentiment scores with the human coded sentiment scores to validate our measure. The two measures were significantly correlated (r = 0.611, *t* (2930) = 41.802, *p* < 0.001), suggesting that our use of the machine learning classifier is reasonable. We focus on results using the machine learning classifier measure of sentiment because this measure is scalable and reproducible, but analogous analyses using the human coded measures of sentiment produce similar results (see Supplementary information), thereby increasing our confidence in the machine classifier and validating our approach.

### Recommendation rating change and sentiment

We then checked whether the sentiment of the peer recommendations influenced whether participants changed their own initial recommendations. Participants changed their initial recommendations 43.12% of the time, primarily in alignment with the sentiment of the peer recommendations; that is, participants changed their initial recommendations to be more positive when they read peer recommendations higher in positivity and vice versa (effect of positivity vs. negativity on the direction of opinion change in a multi-level model accounting for non-independence due to repeated observations from participants and mobile game app: B = 1.038, *t*(2573) = 11.96, *p* < 0.001). In addition, such effects were greater for peer recommendations higher in negativity than positivity, with participants more likely to change their initial recommendation toward that of their peers after reading recommendations higher in negativity (effective of positivity vs. negativity on likelihood to change opinion in a multi-level model accounting for non-independence due to repeated observations from participants and mobile game app: B = − 0.450, *t*(2160) = − 6.928, *p* < 0.001). Thus, both positive and negative peer recommendations significantly and robustly affected participants’ final ratings; further, recommendations that were more negatively framed had the greatest influence in changing the initial recommendation of participants, suggesting that negativity may propagate more strongly than positivity in this context.

### Mean brain activity and sentiment of recommendation

We next examined whether neural activity in the valuation and mentalizing systems was correlated with the sentiment of the peer recommendations. Results indicated that mean activity in the mentalizing regions, but not valuation regions, was greater when participants were considering peer recommendations that were higher in negativity (mentalizing: B = − 0.062, *t*(2929) = − 2.137, *p* = 0.033; valuation: B = 0.016, *t*(1933) = 0.598; *p* = 0.550). Thus, the more that a peer recommendation conveyed negative sentiment about a mobile game app, the greater the engagement of the mentalizing system. By contrast, the sentiment of the reviews was not associated with activity in the valuation system.

### Mean brain activity and recommendation rating change

We next examined whether neural activity in the valuation and mentalizing systems was greater during trials where participants updated their initial recommendation ratings to align with their peers. Increased mean activity in the valuation and mentalizing regions was associated with a significantly higher likelihood that participants changed their ratings to align with the peer recommendation (valuation: B = 0.115, *t*(2765) = 2.696; *p* = 0.007; mentalizing: B = 0.084, *t*(2768) = 2.101, *p* = 0.036). Thus, the more that a written peer recommendation engaged activity in the valuation and mentalizing regions of the brain, the more likely participants were to update their initial recommendations about the mobile game app to align with the peer recommendation. We did not observe any interaction between the sentiment of the review and neural activity in the valuation system in predicting recommendation rating change (see Table 2), suggesting that the value signal was equally indicative of whether a participant would change their initial recommendation to be consistent with the peer recommendation for both positive and negative reviews. In the mentalizing system, however, we observed a marginally significant interaction between the sentiment of the review and brain activity (see Table 3), such that increased response in mentalizing regions to negative recommendations resulted in greater opinion change (simple effect of mentalizing on recommendation rating change for negative peer recommendations: B = 0.129; *t*(1659) = 2.763; *p* = 0.006), but not in response to positive peer recommendations (simple effect of mentalizing on recommendation rating change for positive peer recommendations*:* B = − 0.008; *t*(1075) = − 0.119; *p* = 0.905).

**Table 2:** Predicting participants’ congruent recommendation rating change from mean activity in valuation regions, sentiment of peer recommendations and their interaction (positive coefficients indicate greater change in the direction of the recommendation).

Predictor	*B*	*t*	*df*	*p*
Intercept	0.145	7.088	41.01	< 0.001***
Valuation	0.123	2.897	2764	0.004**
Sentiment	− 0.087	− 7.008	2163	< 0.001***
Valuation*Sentiment	0.027	0.634	2780	0.526

*p < 0.05, **p < 0.01, ***p < 0.001.

**Table 3:** Predicting participants’ congruent recommendation rating change from mean activity in mentalizing regions, sentiment of peer recommendations and their interaction (positive coefficients indicate greater change in the direction of the recommendation).

Predictor	*B*	*t*	*df*	*p*
Intercept	0.144	7.120	40.644	< 0.001***
Mentalizing	0.074	1.865	2766	0.062^†^
Sentiment	− 0.085	− 6.845	2158	< 0.001***
Mentalizing*Sentiment	− 0.065	− 1.669	2785	0.095^†^

^†^*p* < 0.10, *p < 0.05, **p < 0.01, ***p < 0.001.

### Functional connectivity and recommendation rating change

We next examined whether functional connectivity between regions of the brain’s valuation and mentalizing systems was associated with increased or decreased likelihood of recommendation rating change to align with peers. Results using PPI analysis with our valuation regions of interest as a seed indicated that greater connectivity between the brain’s valuation and mentalizing systems was associated with decreased likelihood of recommendation rating change to align with peers (PPI = − 0.003, *t*(32) = − 2.111, *p* = 0.043, where PPI is the parameter estimate of the relationship between activity in the valuation and mentalizing systems during recommendation rating change compared to no recommendation rating change). In other words, recommendation rating change was associated with less correlation in activity between the brain’s valuation and mentalizing systems.

## Discussion

Results of the current study highlight the robust involvement of the brain’s valuation system in tracking and incorporating social influence in situations that are analogous to online recommendations made in the current media environment. Increased brain activity in valuation regions as participants read naturalistic peer recommendations was associated with recommendation rating change to conform with peer opinions. This did not differ by the sentiment of the social influence. Thus, in this context, the brain’s valuation system tracked the value of the peer recommendations, such that increased valuation activity was associated with greater likelihood of recommendation congruent change. Findings also suggest that brain systems that support considering others’ mental states are important in incorporating peer recommendations to inform one’s own recommendations, and that this effect is particularly driven by negatively framed peer recommendations. We also show novel evidence that suggests that decreased connectivity between valuation and mentalizing is associated with recommendation rating change (i.e., increased connectivity between valuation and mentalizing is associated with resistance to peer influence). One possibility is that the brain’s value and mentalizing regions may operate relatively independently or in a less correlated manner when people incorporate others’ opinions to update their own recommendations, whereas negative valuation of peer recommendations might suppress mentalizing.

Using an externally valid paradigm of peer influence on recommendations in the online media context^10^, we found that increased mean activity within the brain’s valuation regions as participants incorporated peer recommendations in real-time is associated with greater recommendation rating change to conform with peer recommendations. This aligns with prior research on peer recommendations in adolescents showing that mean activity in regions of the brain’s valuation system during peer feedback is associated with likelihood to conform to peer influence^10,11^. We extend these findings to suggest that these effects are not specific to adolescents, but also holds in a young adult sample, and in a context with more complex, natural language recommendations (rather than sparser information about peer opinions).

We did not observe a significant interaction between the sentiment of the recommendation and activity in the brain’s valuation system to predict recommendation rating change. This finding suggests that, in this recommendation paradigm, the value signal tracked the value of the peer recommendation when receivers of influence were first exposed to peer opinions and actively made decisions to update their own opinions. In contrast to studies showing that the value signal tracks whether a receiver’s initial opinion is in line with the peer influence, such that greater activity is associated with already agreeing with peers^4,5,8^, we find that greater activity in the value system seems to track likelihood to change opinions to come into alignment with peers^10,11^. This difference may arise from differences in the timing of the peer feedback in different paradigms. In studies where the valuation system was found to track congruence with peer opinion^4,5,8^, receivers’ initial opinions were collected and peer feedback was provided directly after, but receivers’ final opinions were collected in a later session (e.g., 1 h later). In contrast, in studies that have found results consistent to ours (i.e., wherein valuation activity tracks whether or not participants choose to conform to peer influence^10,11^), receivers’ initial opinions were first collected, and then at a later session (e.g., 1 h later), receivers were provided peer feedback and asked about their final opinion immediately after learning the peer feedback. Taken together, these data suggest that the valuation system may serve a different role depending on the relative timing of peer influence and collection of the receivers’ opinions, and hence whether the valuation signal likely tracks the direct valence of the recommendation or the participant’s valuation of the recommendation itself, regardless of the valence. The timing of data collection is particularly relevant to the current online social environment, where online users often read recommendations that are written by others and then immediately post their own recommendations, which is similar to the paradigm we used in the current study. Our results augment a growing body of literature that examine social influence in contexts that more closely resemble online recommendation platforms (e.g., Yelp, Amazon), suggesting the valuation signal tracks the value of the peer recommendation in this context^10,11^.

We found that the mean activity in the brain’s mentalizing regions while participants considered and incorporated peer recommendations was associated with recommendation rating change. These findings corroborate previous research showing that mean activity in regions of the brain’s mentalizing system is implicated in processing of divergent social feedback^10,11^, and that receivers of influence who display greater mean mentalizing activity are also more likely to change their opinion toward that of peer influence^10^.

We observed a marginally significant interaction between the sentiment of the recommendation and activity in the brain’s mentalizing system to predict recommendation rating change. These findings suggest that the brain’s mentalizing system may be recruited more strongly in situations where social consequences are the most salient, such as those that may signal negativity. Our data are consistent with research on negativity bias which suggests that across diverse domains, people are more sensitive to negative than positive information^27^; for instance, negative recommendations have greater impact on consumer behavior than positive recommendations^2^, and negative information more robustly affects formations of social impressions^37,38^. Our data extend these findings to suggest that people show increased neurocognitive and behavioral sensitivity to recommendations that express negativity about an entity, with negative recommendations invoking more thoughts about the social implications of one’s own opinion. Given the importance of social coordination in humans^39,40^, the increased mentalizing response is consistent with the idea that people may find negative recommendations as more socially important or relevant. Indeed, activity in the brain’s social pain and mentalizing regions during social exclusion is associated with greater vulnerability to social influence^23^, and negative information is preferentially propagated over positive information in social contexts^26^. We interpret these findings with caution given that the interaction effect was marginally significant. Nonetheless, our findings are consistent with an account of social influence where negatively framed information may be thought to be more socially salient and lead to greater conformity to social influence.

In a novel contribution, we also examined whether and how valuation and mentalizing regions in the brain might coordinate to respond to peer recommendations. Our data are consistent with the notion that valuation and mentalizing signals operate relatively independently or in a less correlated manner when receivers of influence update their own recommendations in response to peer influence. This is consistent with past research showing that the flexibility of a sub-region of the brains’ valuation system—VMPFC—is associated with greater message-congruent behavior change^41^. Flexibility is an indicator of the degree to which the VMPFC coordinates with different brain networks. Taken together, it is possible that a dynamic VMPFC signal may support the mechanisms necessary to flexibly incorporate the value of new information during decisions to update one’s own opinion or behavior. Our results build on and extend these findings to suggest that a less dynamic VMPFC and value signal (due to being consistently connected with the mentalizing system) is associated with less behavior change. Another possibility is that during exposure to peer recommendations, the value signal tracks the value of the peer recommendation regardless of valence, while the mentalizing system is more responsive under some conditions than others (in this case, negative reviews were more salient in engaging the mentalizing system). A third possibility is that negative valuation of recommendations may actively suppress mentalizing activity (i.e., the two systems may show more coordination when participants do not change their opinion). Additional work that further examines brain network connections will help paint a more complete picture of the neural mechanisms that support social influence.

Combined, our findings contribute insights into the processes that are implicated when people consider recommendations from other consumers when making decisions about a product, as occurs frequently in online shopping environments. Understanding these drivers is particularly important given the tremendous influence that online reviews have on consumer behavior in a wide range of contexts^2,7,42,43^. Further, our study focused on one type of social influence online that shoppers widely engage in: considering recommendations from strangers who have had prior experience with the product. It remains unclear whether our findings would generalize in other contexts, such as when a shopper receives recommendations from a friend, family member, or an intimate partner. The notion of homophily suggests that people are friends with others who are similar to themselves^44^ and become more similar with one another over time due to mutual social influence^45^; thus, one possibility is that the effects that we observed in our study would be even more pronounced when people receive feedback from familiar others compared to strangers. On the other hand, other work suggests that people are less likely to socially conform to friends than strangers ^46^, and that people exert greater physiological synchrony in certain contexts with strangers than friends and romantic partners ^47^. Future work that explicitly tests the associations between the psychological and neural drivers of social influence and the source of the influence would help further clarify between these possibilities.

In conclusion, our data suggest that brain systems that support processing the value of different entities and understanding others’ mental states are associated with recommendation rating change as a result of social influence in a context that mirrors the new media environment. We used real text-based recommendations and tracked how participants’ brains responded to peer feedback in real-time to update their recommendations. Further, we examined whether and how the sentiment of the recommendations interacted with key brain processes to influence recommendation change. We found that the relationship between mentalizing and recommendation rating change was marginally stronger for recommendations that are negative compared to recommendations that are positive, suggesting that valence may be an important factor to consider in future studies of social influence. We further highlight the value of investigating the functional connectivity between these regions in the brain. These results inform how recommendations propagate and the neurocognitive dynamics and features of recommendations that are important to this process.

## Supplementary Information

Supplementary Information.

## Data Availability

The datasets generated during and/or analyzed during the current study are available from the corresponding author on reasonable request.
