# Supplementary material for: Activity in the brain’s valuation and mentalizing networks is associated with propagation of online recommendations
Source: Sci Rep. 2021 May 27;11:11196. doi: 10.1038/s41598-021-90420-2 (PMC8160140; doi:10.1038/s41598-021-90420-2)
Supplement: Supplementary file 1 — Supplementary Information. [file 41598_2021_90420_MOESM1_ESM.pdf]

Activity in the Brain's Valuation and Mentalizing Networks is Associated with Propagation of Online Recommendations

Elisa C. Baek[1a], Matthew Brook O'Donnell[2], Christin Scholz[3], Rui Pei[2], Javier O. Garcia[4,5], Jean M. Vettel[4,5,6], and Emily B. Falk[2,7,8*]

[1]Department of Psychology, University of California, Los Angeles, [2]Annenberg School for Communication, University of Pennsylvania, [3] Amsterdam School of Communication Research, University of Amsterdam, [4] U.S. Army Research Laboratory, [5] Department of Bioengineering, University of Pennsylvania, [6] Department of Psychological Brain Sciences, University of California, Santa Barbara, [7] Department of Psychology, University of Pennsylvania, Philadelphia, PA 19104 [8] Marketing Department, The Wharton School, University of Pennsylvania, Philadelphia, PA 19104

[a] At the time this research was completed, the author was at the Annenberg School for Communication at the University of Pennsylvania. She is now at the University of California, Los Angeles.

*Corresponding Author:

Emily B. Falk, emily.falk@asc.upenn.edu

Annenberg School for Communication, University of Pennsylvania

3620 Walnut St., Philadelphia, PA 19104

Supplementary information

Participant Exclusions

Forty participants (28 females) between the ages of 18 and 24 (M = 20.9, SD = 2.1) were recruited for a single three-hour study appointment, incorporating a one-hour fMRI scan. Participants met standard fMRI eligibility criteria, including being right-handed, not currently taking any psychoactive medications, no history of psychiatric or neurological disorders, not currently pregnant, no metal in their body contraindicated for MRI, and not suffering from claustrophobia. All runs from one participant and run 3 from three participants were excluded due to data corruption. Further, all runs from one participant and one run from one participant were excluded due to excessive movement. This resulted in thirty-eight participants included for analysis, with partial data from four participants.

Additional information about data acquisition

Due to scheduling issues at our scanner center, 33 participants were scanned on a TIM Trio scanner, and the remaining 7 on a Prisma scanner. Models controlling for scanner type showed no significant or meaningful differences from those reported. The fMRI scan for the task of interest was obtained as part of a larger study that included another task. In the current paper, we focus on a single task (the App Rating Task) that spanned 3 runs. Participants completed all 3 runs of the App Rating Task first, and then completed the second task (more information on the second task obtained as part of the same protocol can be found on: https://github.com/cnlab/article_sharing_task).

Due to hypotheses not of interest in the current investigation, each participant read 40 written recommendations from one peer reviewer who had high ego-betweenness centrality and 40 written recommendations from one peer reviewer who had low ego-betweenness centrality.

Main results using human-coded sentiment scores

To validate our machine-learning sentiment classifier, we ran additional models that parallel the main analyses using the human-coded sentiment scores. We found that our results that use human-coded sentiment scores largely support the findings using the machine-learning sentiment classifier that we report in the main manuscript.

Behavioral data analysis. We defined recommendation rating change in an analogous manner to how we defined it in the main manuscript. Accordingly, we defined recommendation rating change as being positive (+1) if the participant changed their initial ratings in the direction of the sentiment of the peer recommendation, negative (-1) if the participant changed their initial ratings away from the sentiment of the peer recommendation, and zero (0) if participants did not change their ratings. For this purpose, peer recommendations were classified into binary categories as either "positive" or "negative" by using the sentiment scores produced by the human coders, which ranged from 0-100 (0 being the most negative and 100 being the most positive). Thus, if the human-coded sentiment scores indicated that the recommendation was more likely to be positive than negative (>50), then it was categorized as positive (and vice versa). Thus, if participants changed their initial recommendation of a "5" to a final recommendation rating of a "3" after reading a peer recommendation that was classified as "positive", then the recommendation rating change was calculated as "+1". Paralleling the method that we used in the main manuscript, to determine the relationship between peer recommendation sentiment scores and participants' recommendation rating change, we ran a multi-level linear regression predicting the participants' recommendation rating change from the sentiment scores of the peer recommendations:

$$\text{recommendation rating change}_{ij} = B_0 + B_1\text{sentiment}_{ij} + \mu_{0i} + \nu_{0j} + \epsilon_{ij},$$

where B_0 is the overall intercept, representing the grand mean across all observations, B_1 is an unstandardized regression coefficient capturing the average slope of the relationship between human-coded sentiment score and recommendation rating change; subscript *i* refers to participant, *j* refers to app, and μ_{0i} and ν_{0j} represent the random errors for the deviation of the mean intercept for each participant and app from the grand mean intercept, respectively, and ε_{ij} is the random error for each app rating within participants. Participants and mobile apps were treated as random effects with intercepts allowed to vary randomly, accounting for non-independence in the data due to repeated measures from each participant.

Recommendation rating change and sentiment (Human-Coded)

Paralleling the main results, participants changed their ratings in alignment with the human-coded sentiment of the peer recommendations (B = 0.012, t (2240) = 18.51; p <.001), and the effects were greater for peer recommendations higher in negativity than positivity (B = -0.003, t (1972) = -7.018, p <.001).

Brain activity and sentiment. We ran analyses examining whether the neural activity in the mentalizing and value also correlated with human coded sentiment scores:

$$\text{mean brain activity}_{ij} = B_0 + B_1\text{sentiment}_{ij} + \mu_{0i} + \nu_{0j} + \epsilon_{ij},$$

where B_0 is the overall intercept, representing the grand mean across all observations, B_1 is an unstandardized regression coefficient capturing the average slope of the relationship between human-coded sentiment score and brain activity; subscript *i* refers to participant, *j* refers to app, and μ_{0i} and ν_{0j} represent the random errors for the deviation of the mean intercept for each participant and app from the grand mean intercept, respectively, and ε_{ij} is the random error for each app rating within participants; “brain activity” represents activity in the target regions of interest, with separate models run for mentalizing and valuation systems.

Paralleling results in the main manuscript, we found that the relationship between mean activity in the mentalizing regions and human coded sentiment scores was marginally significant (B = -0.004, t (2013) = -1.650, p = 0.099), and that the relationship between mean activity in the valuation regions and human coded sentiment scores was not significant (B = 0.002, t (1491) = 0.825, p = 0.409).

Brain activity and recommendation rating change

We next ran analyses examining whether neural activity in the mentalizing and value systems correlated with trials where participants changed their initial ratings toward that of the peers, using the recommendation rating change variable calculated from human coded sentiment scores:

$$\text{recommendation rating change}_{ij} = B_0 + B_1\text{brain activity}_{ij} + \mu_{0i} + \nu_{0j} + \epsilon_{ij},$$

where B_0 is the overall intercept, representing the grand mean across all observations, B_1 is an unstandardized regression coefficient capturing the average slope of the relationship between brain activity and recommendation rating change; subscript i refers to participant, j refers to app, and μ_{0i} and ν_{0j} represent the random errors for the deviation of the mean intercept for each participant and app from the grand mean intercept, respectively, and ε_{ij} is the random error for each app rating within participants; "brain activity" represents activity in the target regions of interest, with separate models run for mentalizing and valuation systems.

We found that mean activity in the mentalizing and value regions was associated with recommendation rating change, though the relationship with mentalizing was marginal (mentalizing: B = 0.075, t (2765) = 1.948, p = 0.052; value: B = 0.102, t (2762) = 2.454, p = 0.014).

We next ran analyses predicting recommendation rating change from the interaction of the human coded sentiment scores and mean brain activity:

recommendation rating $\text{change}_{ij} = B_0 + B_1\text{brain activity} + B_2\text{sentiment} + B_3\text{brain activity*sentiment} + \epsilon_{ij}$,

where B_0 is the overall intercept, representing the grand mean across all observations, B_1 is an unstandardized regression coefficient capturing the average slope of the relationship between brain activity and recommendation rating change, B_2 is an unstandardized regression coefficient capturing the average slope of the relationship between human-coded sentiment scores and recommendation rating change, B_3 is an unstandardized regression coefficient capturing the average slope of the interaction effect of brain activity and sentiment on recommendation rating change; subscript i refers to participant, j refers to app, and μ_{0i} and ν_{0j} represent the random errors for the deviation of the mean intercept for each participant and app from the grand mean intercept, respectively, and ε_{ij} is the random error for each app rating within participants; "brain activity" represents activity in the target regions of interest, with separate models run for mentalizing and valuation systems).

We found a directional trend of an interaction between the sentiment of the review and neural activity in the mentalizing system in predicting recommendation rating change (see Table S1 below), but not an interaction between the sentiment of the review and neural activity in the valuation system in predicting recommendation rating change (see Table S2 below).

Table S1. Predicting participants' congruent recommendation rating change from mean activity in mentalizing regions, human-coded sentiment of peer recommendations and their interaction (positive coefficients indicate greater change in the direction of the recommendation).

Predictor	*B*	*t*	*df*	*p*
Intercept	0.199	8.513	39.22	<.001***
Mentalizing	0.067	1.744	2763	0.081†
Sentiment	-0.084	-6.917	1982	<.001***
Mentalizing*Sentiment	-0.061	-1.504	2782	0.133

Table S2. Predicting participants' congruent recommendation rating change from mean activity in valuation regions, human-coded sentiment of peer recommendations and their interaction (positive coefficients indicate greater change in the direction of the recommendation).

Predictor	*B*	*t*	*df*	*p*
Intercept	0.199	8.465	39.45	<.001***
Valuation	0.108	2.616	2762	0.009**
Sentiment	-0.086	-7.092	1988	<.001***
Valuation*Sentiment	0.033	0.769	2773	0.442

Main results with subregions of the mentalizing and valuation ROIs

To complement our main results that extracted percent signal change in all the voxels of our mentalizing and valuation ROIs (as defined from Neurosynth), we ran the same analyses using the subregions in each of the ROIs. We used regions.connected_regions from the nilearn

package in Python 3 [42] to extract 10 contiguous clusters from the mentalizing network and 2 contiguous clusters from the valuation network. We then repeated the main analyses as described in the Methods section of the main manuscript. We first examined whether neural activity in each of our subregions was associated with the sentiment of the peer recommendations (Tables S3 and S4).

Table S3. Predicting Brain Activity in Subregions of the Mentalizing ROI from the Sentiment of the Recommendations

Brain Region (dependent variable)	*B* *(Sentiment)*	*t*	*df*	*P*
Right temporal lobe	-0.072	-2.246	2916	0.025*
Right temporoparietal junction	-0.055	-1.919	2244	0.055†
Right cerebellum	-0.062	-1.568	2292	0.117
Right supplementary motor area	-0.023	-0.635	2930	0.526
Precuneus	-0.021	-0.582	2930	0.561
Dorsomedial prefrontal cortex	-0.082	-2.244	2304	0.025*
Ventromedial prefrontal cortex	-0.025	-0.659	2808	0.510
Left temporal lobe	-0.049	-1.761	1996	0.078†
Left cerebellum	-0.062	-1.543	1811	0.123
Left temporoparietal junction	-0.078	-2.537	2835	0.011*

Note: Each row of the table represents a distinct model, wherein each subregion of the mentalizing ROI is the dependent variable in each model, respectively:

$$\text{mean brain activity}_{ij} = B_0 + B_1\text{sentiment}_{ij} + \mu_{0i} + \nu_{0j} + \epsilon_{ij},$$

where B_0 is the overall intercept, representing the grand mean across all observations, B_1 is an unstandardized regression coefficient capturing the average slope of the relationship between sentiment and brain activity; subscript *i* refers to participant, *j* refers to app, and μ_{0i} and ν_{0j} represent the random errors for the deviation of the mean intercept for each participant and app from the grand mean intercept, respectively, and ε_{ij} is the random error for each app rating within participants; "brain activity" represents activity in the target ROI.

Table S4. Predicting Brain Activity in Subregions of the Valuation ROI from the Sentiment of the Recommendations

Brain Region (dependent variable)	*B* *(Sentiment)*	*t*	*df*	*P*
Ventromedial Prefrontal Cortex	0.015	0.442	1675	0.684
Ventral Striatum	0.031	1.155	2875	0.248

Note: Each row of the table represents a distinct model, wherein each subregion of the valuation ROI is the dependent variable in each model, respectively:

$$\text{mean brain activity}_{ij} = B_0 + B_1\text{sentiment}_{ij} + \mu_{0i} + \nu_{0j} + \epsilon_{ij},$$

where B_0 is the overall intercept, representing the grand mean across all observations, B_1 is an unstandardized regression coefficient capturing the average slope of the relationship between sentiment and brain activity; subscript *i* refers to participant, *j* refers to app, and μ_{0i} and ν_{0j} represent the random errors for the deviation of the mean intercept for each participant and app from the grand mean intercept, respectively, and ε_{ij} is the random error for each app rating within participants; "brain activity" represents activity in the target ROI.

We next examined the relationship between subregions of the mentalizing and valuation ROIs and recommendation rating change (see Tables S5 and S6).

Table S5. Predicting Recommendation Rating Change by Subregions of the Mentalizing ROI

Brain Region	*B*	*t*	*df*	*P*
Right temporal lobe	0.034	0.953	2766	0.341
Right temporoparietal junction	0.053	1.302	2770	0.193
Right cerebellum	0.034	1.160	2770	0.246
Right supplementary motor area	0.042	1.277	2763	0.202
Precuneus	0.070	2.172	2766	0.03*
Dorsomedial prefrontal cortex	0.068	2.154	2768	0.031*
Ventromedial prefrontal cortex	0.071	2.374	2766	0.018*
Left temporal lobe	0.055	1.322	2770	0.186
Left cerebellum	0.031	1.059	2766	0.29
Left temporoparietal junction	0.047	1.236	2767	0.216

Note: Each row of the table represents a distinct model, wherein each subregion of the mentalizing ROI is the independent variable in each model, respectively:

$$\text{recommendation rating change}_{ij} = B_0 + B_1\text{brain activity}_{ij} + \mu_{0i} + \nu_{0j} + \epsilon_{ij},$$

where B_0 is the overall intercept, representing the grand mean across all observations, B_1 is an unstandardized regression coefficient capturing the average slope of the relationship between brain activity and recommendation rating change; subscript i refers to participant, j refers to app, and μ_{0i} and ν_{0j} represent the random errors for the deviation of the mean intercept for each participant and app from the grand mean intercept, respectively, and ε_{ij} is the random

error for each app rating within participants; "brain activity" represents activity in the target ROI).

Table S6. Predicting Recommendation Rating Change by Subregions of the Valuation ROI

Brain Region (dependent variable)	*B* *(Sentiment)*	*t*	*df*	*P*
Ventromedial Prefrontal Cortex	0.093	2.694	2766	0.007**
Ventral Striatum	0.085	1.961	2762	0.049*

Note: Each row of the table represents a distinct model, wherein each subregion of the valuation ROI is the independent variable in each model, respectively:

$$\text{recommendation rating change}_{ij} = B_0 + B_1\text{brain activity}_{ij} + \mu_{0i} + \nu_{0j} + \epsilon_{ij},$$

where B_0 is the overall intercept, representing the grand mean across all observations, B_1 is an unstandardized regression coefficient capturing the average slope of the relationship between brain activity and recommendation rating change; subscript *i* refers to participant, *j* refers to app, and μ_{0i} and ν_{0j} represent the random errors for the deviation of the mean intercept for each participant and app from the grand mean intercept, respectively, and ε_{ij} is the random error for each app rating within participants; "brain activity" represents activity in the target ROI).
